# Zinc attenuates ferroptosis and promotes functional recovery in contusion spinal cord injury by activating Nrf2/GPX4 defense pathway

**DOI:** 10.1111/cns.13657

**Published:** 2021-05-05

**Authors:** Ming‐hao Ge, He Tian, Liang Mao, Dao‐yong Li, Jia‐quan Lin, Heng‐shuo Hu, Shuo‐cheng Huang, Chuan‐jie Zhang, Xi‐fan Mei

**Affiliations:** ^1^ Department of Orthopedics The First Affiliated Hospital of Jinzhou Medical University Jinzhou China; ^2^ Department of Histology and Embryology Jinzhou Medical University Jinzhou China; ^3^ Department of Oncology The First Affiliated Hospital of Jinzhou Medical University Jinzhou China

**Keywords:** ferroptosis, inflammation, nuclear factor E2, spinal cord injury, zinc

## Abstract

**Aim:**

Spinal cord injury (SCI) involves multiple pathological processes. Ferroptosis has been shown to play a critical role in the injury process. We wanted to explore whether zinc can inhibit ferroptosis, reduce inflammation, and then exert a neuroprotective effect.

**Methods:**

The Alice method was used to establish a spinal cord injury model. The Basso Mouse Scale (BMS), Nissl staining, hematoxylin‐eosin staining, and immunofluorescence analysis were used to investigate the protective effect of zinc on neurons on spinal cord neurons and the recovery of motor function. The regulation of the nuclear factor E2/heme oxygenase‐1 (NRF2/HO‐1) pathway was assessed, the levels of essential ferroptosis proteins were measured, and the changes in mitochondria were confirmed by transmission electron microscopy and 5,5′,6,6′‐tetrachloro‐1,1′,3,3′‐tetraethyl‐imidacarbocyanine iodide (JC‐1) staining. In vitro experiments using VSC4.1 (spinal cord anterior horn motor neuroma cell line), 4‐hydroxynonenal (4HNE), reactive oxygen species (ROS), superoxide dismutase (SOD), malondialdehyde (MDA), glutathione (GSH), lipid peroxides, and finally the levels of inflammatory factors were detected to assess the effect of zinc.

**Results:**

Zinc reversed behavioral and structural changes after SCI. Zinc increased the expression of NRF2/HO‐1, thereby increasing the content of glutathione peroxidase 4 (GPX4), SOD, and GHS and reducing the levels of lipid peroxides, MDA, and ROS. Zinc also rescued injured mitochondria and effectively reduced spinal cord injury and the levels of inflammatory factors, and the NRF2 inhibitor Brusatol reversed the effects of zinc.

**Conclusion:**

Zinc promoted the degradation of oxidative stress products and lipid peroxides through the NRF2/HO‐1 and GPX4 signaling pathways to inhibit ferroptosis in neurons.

## Graphical abstract

Mechanisms underlying the repair of spinal cord injury through inhibiting ferroptosis with zinc.

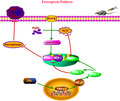

## 1 INTRODUCTION

Spinal cord injury is a severe traumatic central nervous system injury with high mortality and high disability that places severe economic burdens on individuals and society.^1^, ^2^, ^3^ Most spinal cord injuries are associated with physiological and biochemical changes and structural abnormalities. The pathophysiological manifestations of spinal cord injury include primary and secondary injuries. It is usually the primary injury that determines the consequent injury and, to a large extent, the future recovery of the spinal cord.^4^ Clinical studies have shown that traumatic spinal cord injury has different pathological characteristics in different time course. Studies have found that spinal cord swelling, cell membrane, or vascular rupture leads to ischemia in patients with traumatic spinal cord injury in 0–2 h, leading to gray matter hemorrhagic necrosis and peripheral white matter hemorrhage. However, microglia were activated at the same time, which could release inflammatory cytokines such as IL‐1 β, IL‐6, and TNF‐α 2 h to 2 days, and continuous spinal cord hemorrhage caused local ischemia, axonal swelling, neuron, and oligodendrocyte death. At 2–14 days, macrophages infiltrated, astrocytes proliferated, and scars were produced. The glial scar continued to form from 14 days to 16 days. After 16 days is the chronic stage, the lesion site is stable and mature, when there are cystic vacuoles and spinal cord softening is the final stage, namely spinal cord necrosis. This process includes various pathological processes, such as ferroptosis, necrosis, apoptosis, and inflammation.^5^, ^6^ Bleeding occurs at the injured site during the initial stage of injury. A large number of blood cells and hemoglobin are destroyed and degraded, resulting in a sharp increase in the level of iron ions in the injured area. Additionally, the tissue at the injured site is necrotic and produces many reactive oxygen species and oxidative stress products.^7^, ^8^, ^9^, ^10^ The content of saturated fatty acids is very high. Finally, due to iron and lipid supersaturation, the production of lipid peroxides suddenly increases, and iron catalyzes enzyme‐mediated programmed cell death.^11^, ^12^ Numerous biochemical reactions and physiological changes in the body involve zinc. Zinc is closely linked to the growth and maturation of neurons and energy metabolism.^13^, ^14^ Relevant studies have shown that an appropriate amount of zinc can have antioxidative, antiapoptotic, and immunomodulatory effects.^15^ Our previous study showed that zinc can regulate NRF2/HO‐1 to a certain extent. This pathway can have a role in the accumulation of oxidative stress products and reactive oxygen species.^10^, ^16^, ^17^

GPX4 is the principal regulator of this kind of programmed death. Studies indicate that nuclear factor E2 (NRF2) is one of the most critical factors in regulating the antioxidant response. The GPX4 gene is among the genes regulated by NRF2.^10^, ^18^, ^19^, ^20^, ^21^ Therefore, GPX4 is also crucial to the regulation of ferroptosis.^22^ Regarding the regulation of GPX4 to reduce the accumulation of lipid peroxidation products, this kind of programmed death has not been demonstrated in the mechanism of spinal cord injury, so we chose a commonly used antioxidant, zinc, to inhibit ferroptosis after spinal cord injury.^16^ In this experiment, we found that the expression of NRF2/HO‐1 was significantly increased by zinc. NRF2/HO‐1 thereby regulated GPX4 and 4HNE (lipid peroxide markers) to reduce the generation of oxidative stress products and the accumulation of lipid peroxides. We confirmed that zinc regulated ferroptosis via the NRF2/HO‐1 pathway, decreased the inflammatory infiltration of the injured site, and ultimately promoted neuronal survival to alleviate spinal cord injury.

## 2 METHODS

### 2.1 Animal

A total of 160 female C57BL/6 J Nifdc mice (20–25 g, 8 weeks old) were purchased from Beijing Weitong Lihua Experimental Animal Technology Co., Ltd. (license no: SCXK (Jing) 2016–0006; Beijing, China). To exclude sex differences, all experimental animals were female mice. The mice were housed in a specific pathogen‐free laboratory center at Jinzhou Medical University in standard cages (five mice per cage) with a 12‐h/12‐h light/dark cycle. The mice had free access to food and water, and their environment was maintained at a temperature of 22–24°C. Furthermore, the mice were allowed to adapt to the environment for one week before the experiment.

### 2.2 Spinal cord injury and treatment of drugs

The experimental method was approved by the Animal Protection and Use Committee of Jinzhou Medical University. The animal data were reported following the ARRIVE 2.0 guidelines.^23^ Mice were randomly divided into four groups: sham group, SCI group, SCI + ZnG injection group (SCI + ZnG), and SCI + ZnG plus Brusatol injection group (Brusatol). To induce SCI, the animals were deeply anesthetized by intrapulmonary injection of an anesthesia with 1% sodium pentobarbital (50 mg/kg; P‐010, Sigma‐Aldrich, USA).^16^ Each mouse was placed in the prone position on the operating table, the spine was fully exposed, and then laminectomy was performed on the T9/T10 vertebrae. The spinal cord was fully exposed, and a modified impactor (diameter: 2 mm, weight: 12.5 g, height: 1 cm) was utilized to strike the spinal cord at T9/T10, causing a moderate contusion.^24^ The sham group underwent the same procedure without spinal cord contusion, and the skin was sutured after hemostasis. During the entire experiment, the mice were observed daily for health, activity in the cage, and infection. Artificial bladder massage was performed twice a day for urination during the first week after surgery and then once a day until the mice resumed urination on their own. Mice in the SCI + ZnG group and SCI + ZnG + Brusatol group were injected intraperitoneally with ZnG (Biotopped, China) or Brusatol (25 mg/mL; #SML1868, Sigma‐Aldrich, USA) at a dose of 30 mg/kg or 2 mg/kg in 0.1 mL vehicle [DMSO (D4540, Sigma‐Aldrich, USA) and 0.9% NaCl (MB2471, Meilunbio, China), 1:3] two hours after the successful establishment of the model and were injected once a day until the third day.^25^, ^26^ The mice in the SCI group were injected intraperitoneally with an equivalent dose of isotonic glucose in 0.1 mL vehicle (DMSO and 0.9% NaCl, 1:3) two hours after successful modeling. They were injected once a day until the third day. The mice in the sham group were injected intraperitoneally with 0.1 mL of vehicle (DMSO and 0.9% NaCl, 1:3) two hours after successful modeling and were injected once a day until the third day.

### 2.3 Behavioral score

According to the BMS mouse hindlimb movement scoring method proposed by Basso et al^27^, the mice were evaluated by exercise behavioral response. The scores range from 0 to 9 points (0 points mean complete paralysis and 9 points mean completely normal) and are based on observation of the frequency, range of motion, coordination, rear ankle joint activities, touch of the soles of the feet and insteps, position of the feet, stability of the trunk, and position of the tail. The operators were blinded to the experimental conditions of the mice, and the assessments were repeated three times and recorded immediately. All personnel were professionally trained. The mice were evaluated at one hour before surgery, three days after surgery, and, beginning seven days later, weekly at the same time every week until the end of the sixth week.

### 2.4 Tissue preparation

At 3 and 28 days after surgery, each group of animals underwent deep anesthetic and cardiac perfusion first with 0.9% sodium chloride and then with 4% polyformaldehyde until the mouse body became stiff and rigid. Mouse spinal cord samples 0.5 cm in length were removed from the upper and lower areas of the injury, washed with PBS, and placed in 4% PFA for fixation. After 48 h of fixation, the tissue was transferred to a 4% paraformaldehyde solution containing 10% sucrose until the tissue sank to the bottom; then, it was transferred to a 4% paraformaldehyde solution containing 20% sucrose until it sank to the bottom. Finally, the tissue was transferred to a 4% paraformaldehyde solution containing 30% sucrose. A cryostat tissue microtome was used to cut the spinal cord area 3 mm above and below the injury point to obtain frozen sections (thickness: 15 μm). Tissue was fixed for 48 h and rinsed with running water for 4 h. After gradient dehydration, the tissue was soaked in xylene and then embedded in paraffin wax. An embedding machine was used to perform paraffin embedding, and finally slices were cut using a paraffin microtome to obtain paraffin sections (thickness: 7 μm). The paraffin sections were maintained at room temperature, and the frozen sections were stored at −80°C.

### 2.5 Hematoxylin‐eosin staining and Nissl staining

Paraffin sections were soaked in water for 5 min, stained with hematoxylin (Solarbio, Beijing, China) aqueous solution for 5 min, rinsed with thrice‐distilled water, differentiated in ammonia and acidic water for 30 s, and then soaked in distilled water for 15 min. Eosin (Solarbio, Beijing, China) staining was performed for another 2 min, the sections were rinsed with thrice‐distilled water, gradient alcohol dehydration and xylene clearing were performed, and finally neutral gum was used to mount the slides to observe the tissue cavity and cell number in each group. As above, after dewaxing, sections were soaked in thrice‐distilled water for 5 min and stained with methyl violet staining solution for 10 min. The slides were rinsed with thrice‐distilled water, differentiated with Nissl differentiation (Beyotime, Beijing, China) solution for 5 s, dehydrated in gradient alcohol solutions and cleared in xylene, and finally placed on slides with neutral gum. Finally, an optical microscope was utilized to observe the changes in Nissl body number in the anterior horn of the spinal cord sliced at the same position.

### 2.6 qRT‐PCR

After killing the mouse with sufficient anesthetic, 1.5‐cm‐long tissue samples were taken from the mouse's upper and lower spinal cord injury areas, total RNA was extracted with TRIzol reagent (Ambion, Foster City, CA, USA), and the RNA concentration was determined. Five micrograms of total RNA was used for reverse transcription to synthesize cDNA (Promega, Fitchburg, WI, USA), and then qRT‐PCR was performed using SYBR Green (Promega, Fitchburg, WI, USA). The cDNA sample was amplified under the following conditions: 95°C for 3 min and then 95°C for 15 s and 60°C for 15 s for 40 cycles. Target gene expression levels were normalized relative to the housekeeping gene ribosomal protein S18, and the target gene expression of the experimental group was compared with the corresponding target gene expression of the control group using the (1 + e) ΔΔCT method. The primer sequences were obtained from Comate Bioscience Co., Ltd. of China. The primers used in the study are listed above in Table 1 (*n* = 6 mice/group).

**TABLE 1:** Primer sequences used in qRT‐PCR

Gene	Primer sequence (5′–3′)	Product size (bp)
NRF2	Forward primer: AGGTTGCCCACATTCCCAAA Reverse primer: ACGTAGCCGAAGAAACCTCA	108
HO−1	Forward primer: CTCCTCTCGAGCGTCATCAG Reverse primer: ATCCTGGGGCATGCTGTC	105
Rps18	Forward primer: CCTGAGAAGTTCCAGCACATTTTG Reverse primer: CCTCCGTGAGTTCTCCAGCC	169

### 2.7 Western blot

We used the same method as before to perform Western blotting.^28^, ^29^ The primary antibodies we used were anti‐NRF2 (1:1000, Abcam, ab31163); anti‐HO‐1 (1:1000, Abcam, ab68477); anti‐GPX4 (1:1000, Affinity Biosciences, DF6701); anti‐4HNE (1:5000, Abcam, ab45545); anti‐TNF‐α (1:1000, Affinity Biosciences, AF7014); anti‐IL‐6 (1:1000, Affinity Biosciences, DF6087); anti‐IL‐1β (1:1000, Affinity Biosciences, AF5103); and anti‐ICAM‐1 (1:1000, Affinity Biosciences, AF6088) antibodies. BeyoECL Plus (Beyotime, Beijing, China) was used for developing immunoblots, and a Tanon 2500R Gel Imaging System (Tanon, Shanghai, China) was used to take pictures and store protein bands. The band intensity was quantified by ImageJ 1.39 V software (*n* = 6 mice/group).

### 2.8 Immunofluorescence staining

Mouse frozen sections were placed at room temperature to thaw for 1 h, washed with 1× PBS 3 times, incubated with Triton X‐100 (0.3%) for 10 min at room temperature, and washed three times with PBS for 5 min per wash. The sections were incubated with BSA at room temperature for 2 h, and then the sections were incubated with primary antibodies overnight at room temperature. On the second day, the unbound primary antibody was washed away with 1× PBS 3 times, and then the sections were incubated with secondary antibodies for two hours at room temperature. Alexa Fluor 488 goat anti‐rabbit IgG or Alexa Fluor 594 goat anti‐mouse IgG (1:250, Thermo Fisher Science, A‐11034/A‐11005) was used. Finally, the sections were incubated with DAPI for 10 minutes at room temperature. A fluorescence microscope was used to observe the tissue sections. ImageJ was used to examine the fluorescence intensity. The method used for immunofluorescence analysis of cells was the same as that used for tissue sections. VSC4.1 were seeded into a 24‐well plate. After the cells were treated, they were washed three times with 1× PBS for five minutes per wash, Triton X‐100 (0.1%) was used to permeabilize the cells, and the cells were washed three times with 1× PBS for five per wash. The cells were blocked with goat serum at room temperature for two hours. Then, the cells were incubated with the primary and secondary antibodies. The primary antibodies used were anti‐Nrf2 (1:1000, Abcam, ab31163); anti‐HO‐1 (1:1000, Abcam, ab68477); anti‐GPX4 (1:1000, Affinity Biosciences, DF6701); anti‐TNF‐α (1:1000, Affinity Biosciences, AF7014); anti‐IL‐6 (1:1000, Affinity Biosciences, DF6087); anti‐IL‐1β (1:1000, Affinity Biosciences, AF5103); and anti‐ICAM‐1(1:1000, Affinity Biosciences, AF6088).

### 2.9 Transmission electron microscopy

Three days after treatment with zinc gluconate, the mice were given a sufficient amount of anesthetic, and the mice were fixed by injecting 40 mL of normal saline and then 50 mL of 3% glutaraldehyde fixative into the left heart. Finally, the injured tissue was removed with a scalpel. Spinal cord tissue samples 0.5 cm in length were collected, and the tissue was stored in glutaraldehyde fixative. The tissue samples that disappeared after the fixation time exceeded 24 h were removed, rinsed with 1× PBS three times for five minutes per wash, fixed with 1% osmium acid for 2 h, and then rinsed with 1× PBS three times for 5 min wash. Then, the samples were dehydrated in an ethanol gradient (50%, 70%, 80%, 90%, 95%; samples were dehydrated at each concentration for 15 min). Finally, the samples were treated with pure acetone for 20 min, the embedding agent was infiltrated with a gradient, and a mixture of embedding agent and acetone (1:1) was used to treat the sample for one hour. The sample was treated with a mixture of embedding agent and acetone (3:1) for three hours and permeated with pure embedding agent overnight. The osmotic‐treated samples were placed in 0.5‐mL Eppendorf tubes, embedded, and heated at 70°C overnight. Spinal cord tissue samples from each group were cut into ultrathin sections, placed on a copper mesh, and stained with 2% uranyl acetate for 20 min and 0.04% lead citrate for 10 min, and the ultrastructure was observed by transmission electron microscopy (Philips Tecnai 10, Amsterdam, The Netherlands) (*n* = 6 mice/group).

### 2.10 Cell culture

VSC4.1 motor neuron cells were cultured at 37°C and 5% CO2 in DMEM (Gibco, Grand Island, NY, USA) containing 10% FBS and 0.4% penicillin‐streptomycin (Gibco, Grand Island, NY, USA) to simulate the oxidative stress response to neuronal injury in vivo. VSC4.1 were grouped as the vehicle group, the H202 group and H2O2 + ZnG group, and the H2O2 + ZnG + Brusatol group. VSC4.1 were treated with H2O2 (60 μmol/L) for 3 h, while the zinc gluconate treatment group was treated with H2O2 (60 μmol/L) + ZnG (90 μmol/L). The treatment time was 3 h, and the NRF2 signaling protein inhibitor Brusatol (25 mg/mL; #SML1868, Sigma‐Aldrich) was administered (0.3 μmol/L). The treatment time was 3 h. The vehicle group was cultured in DMEM, and the other processing factors were precisely the same.

### 2.11 Cell viability assay

The cytotoxicity of each drug to VSC4.1 was evaluated using MTT assays and cell count analyses. First, VSC4.1 were seeded into a 96‐well plate (5–10×10^4^ cells/well). The cells were treated with different concentrations of drugs and cultured at 5% CO2 and 37°C for 3 h. Then, 5 mg/mL MTT was added and incubated for 4 h. Then, the supernatant was discarded, 150 μL dimethyl sulfoxide (DMSO) was added, and the cells were incubated with shaking for 10 min. Absorbance in each well was measured at 490 nm with a microplate reader (Varioskan Flash, Thermo Scientific, Waltham, MA, USA).

### 2.12 Mitochondrial membrane potential measurement

A JC‐1 kit (#C2005, Beyotime Institute of Biotechnology, Nantong, Jiangsu, China) was used to detect the membrane potential of VSC4.1. VSC4.1 were seeded in a 24‐well plate and stained with JC‐1 via incubation in the working solution for 20 min, and then a fluorescence microscope was used to observe the fluorescence intensity of each group of cells.

### 2.13 Lipid peroxidation assay

Lipid peroxidation was detected by BODIPY™ 581/591 C11 (C10445; Thermo Fisher). Moreover, BODIPY™ 581/591 C11 (2 µmol/L) was directly incubated with adherent cells for 30 min at 37°C; confocal microscopy was used to detect the fluorescence.

### 2.14 ROS assay

A Reactive Oxygen Species Assay Kit (#S0033, Beyotime Institute of Biotechnology, Nantong, Jiangsu, China) was used to detect the ROS content of each group after VSC4.1 were processed. DCFH‐DA and VSC4.1 were incubated in a 37℃ incubator for 20 min, the cells were observed under a fluorescence microscope, and finally the cellular ROS fluorescence intensity was measured.

### 2.15 SOD and MDA Assays

The MDA Content Detection Kit (#BC0020, Beyotime Institute of Biotechnology, Nantong, Jiangsu, China) and SOD Activity Detection Kit (#BC0175, Beyotime Institute of Biotechnology, Nantong, Jiangsu, China) were used to detect the MDA content and SOD contents of the mouse spinal cord, respectively. First, 1.5‐cm‐long spinal cord samples were collected from each group of mice, and then the procedure was performed according to the instructions of the kits to calculate the content of each sample (*n* = 6 mice/group).

### 2.16 Statistical analysis

IBM SPSS software version 18.0 was used for data analysis. The Shapiro‐Wilk test was used to assess data distribution. All data are expressed as the mean ± standard deviation. If there were more than two sets of data, one‐way analysis of variance (ANOVA) was used, and then the Bonferroni post hoc test was performed if the variances were equal. The Mann‐Whitney U‐test was used instead when the variance was not equal. The BMS score was analyzed with repeated measures and two‐way ANOVA, and then Tukey's post hoc test was conducted to compare the differences between groups on the same day. *p *< 0.05 was considered statistically significant.

## 3 RESULT

### 3.1 Zinc can improve the behavior and organization of spinal cord injury

**FIGURE 1:**
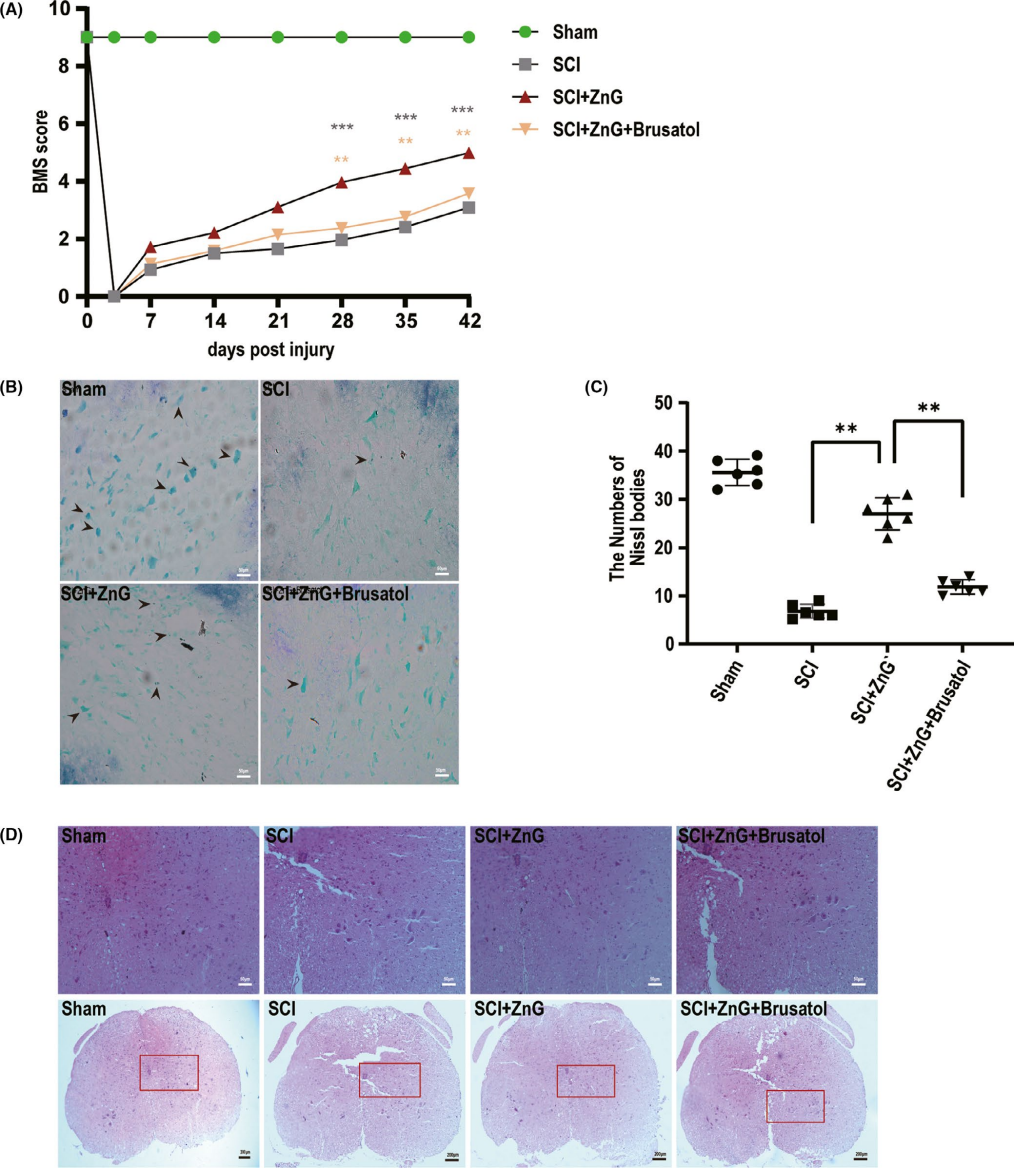
Zinc can improve the behavior and organization of spinal cord injury. (A) Use BMS score to evaluate the recovery of motor function in sham group, SCI group, SCI + ZnG group, and SCI + ZnG + Brusatol group, respectively, in 3, 7, 14, 21, 28, 35, and 42 days (*n* = 10, all the data are expressed as means ± SD, two‐way ANOVA followed by Tukey's post hoc test was applied). (B–C) Nissl staining to observe the number of motor neurons in the spinal cord anterior horn, and the results were analyzed. Arrows indicate neurons containing Nissl bodies (*n* = 6, scale bar = 50 µm, all the data are expressed as means ± SD, two‐way ANOVA followed by Tukey's post hoc test was applied). (D) Image of representative HE‐stained transverse sections of spinal cords from sham group, SCI group and ZnG group, and Brusatol group (*n* = 6, scale bar =50 µm). * means *p* < 0.05, ** means *p* < 0.01, and *** means *p* < 0.001

The experiment detected the effects of zinc on mouse exercise recovery and was measured by determining BMS scores on days 3, 7, 14, 21, 28, 35, and 42 after SCI. Within three days after SCI, the scores of each group immediately dropped to 0 points, and the scores began to increase over time, but the scores of each group were still lower than those of the sham group (Figure 1A). The scores of the SCI + vehicle group, the SCI + ZnG group, and the SCI + ZnG + Brusatol group all increased, but the SCI + ZnG group showed a greater increase than the other two groups. Nevertheless, there was no significant difference. After 28 days and especially at 35 days after SCI, the SCI + ZnG group was significantly different from the SCI + vehicle group and the SCI + ZnG + Brusatol group (*p *< 0.01). ZnG group had a more positive effect on the recovery of motor function than the control group and Brusatol group. Next, Nissl staining showed that the number of neurons in the anterior horn of the spinal cord of SCI + ZnG group mice was greater than that of SCI + vehicle and SCI + ZnG + Brusatol group mice (SCI + ZnG vs. SCI + vehicle, *p* < 0.001; SCI + ZnG vs. SCI + ZnG + Brusatol, *p* < 0.001; Figure 1B, C). HE staining showed that at four weeks after spinal cord injury, the cavities and tissue damage in the SCI + ZnG group were significantly lighter than those in the SCI + vehicle and SCI + ZnG + Brusatol groups. The zinc group showed reduced cavities and tissue edema of the injured spinal cord (Figure 1D).

### 3.2 Zinc can protect neurons and inhibit spinal cord ferroptosis by increasing the expression of NRF2 and HO‐1

**FIGURE 2:**
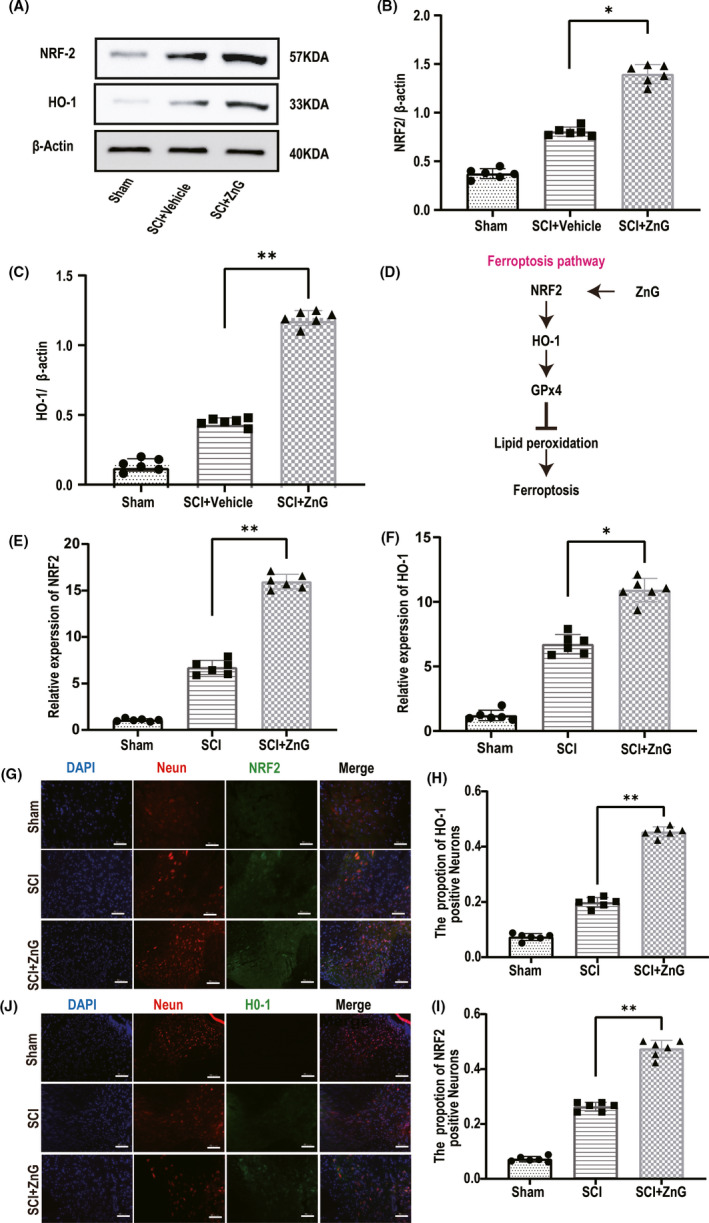
Zinc can protect neurons and inhibit spinal cord ferroptosis by increasing the expression of NRF2 and HO‐1. (A) The expressions of NRF2 and HO‐1 were evaluated at 3 d post‐SCI by Western blotting. (B‐C) Quantification of NRF2 and HO‐1 expressions (*n* = 6, all the data are expressed as means ± SD, two‐way ANOVA followed by Tukey's post hoc test was applied). (D) The schematic diagram of the ferroptosis pathway. (E–F) Quantitative real‐time PCR (qRT‐PCR; *n* = 6 per group, one‐way analysis of variance (ANOVA) followed by Bonferroni's post hoc test). (G–J) Immunofluorescence staining was used to detect the level of NRF2/HO‐1 from each group. (H, I) Statistical analysis of immunofluorescence staining for positive expression of NRF2/HO‐1 in nerve cells from each group (*n* = 6, scale bar = 50 µm, all the data are expressed as means ± SD, two‐way ANOVA followed by Tukey's post hoc test was applied). * means *p* < 0.05, ** means *p* < 0.01, and *** means *p* < 0.001

The results indicated that the expression of NRF2 in the SCI + ZnG group was significantly higher than that in the SCI group. The increase in the expression of NRF2 in the SCI + vehicle group compared with the sham group, as shown in Western blotting (*p* < 0.01) and qRT‐PCR (SCI VS. SCI + ZnG, *p* < 0.01; Figure 2A, B, E), should manifest as a stress response after spinal cord injury. The expression level of HO‐1 in the SCI + ZnG group was also significantly higher than that in the SCI + vehicle group (Figure 2A, C, F). Next, immunofluorescence also showed that the expression of NRF2 and HO‐1 in spinal cord neurons after zinc treatment was significantly increased compared with that in SCI + vehicle‐treated spinal cord neurons (Figure 2G–J). Due to the increase in the expression of NRF2, it naturally leads to the increase in the expression of HO‐1. ZnG inhibits lipid peroxidation by regulating the expression of NRF2, HO‐1, and Gpx4, thereby inhibiting iron death (Figure 2D).

### 3.3 VSC4.1 can also increase the expression of NEF2/HO‐1 after zinc treatment to prevent H202

**FIGURE 3:**
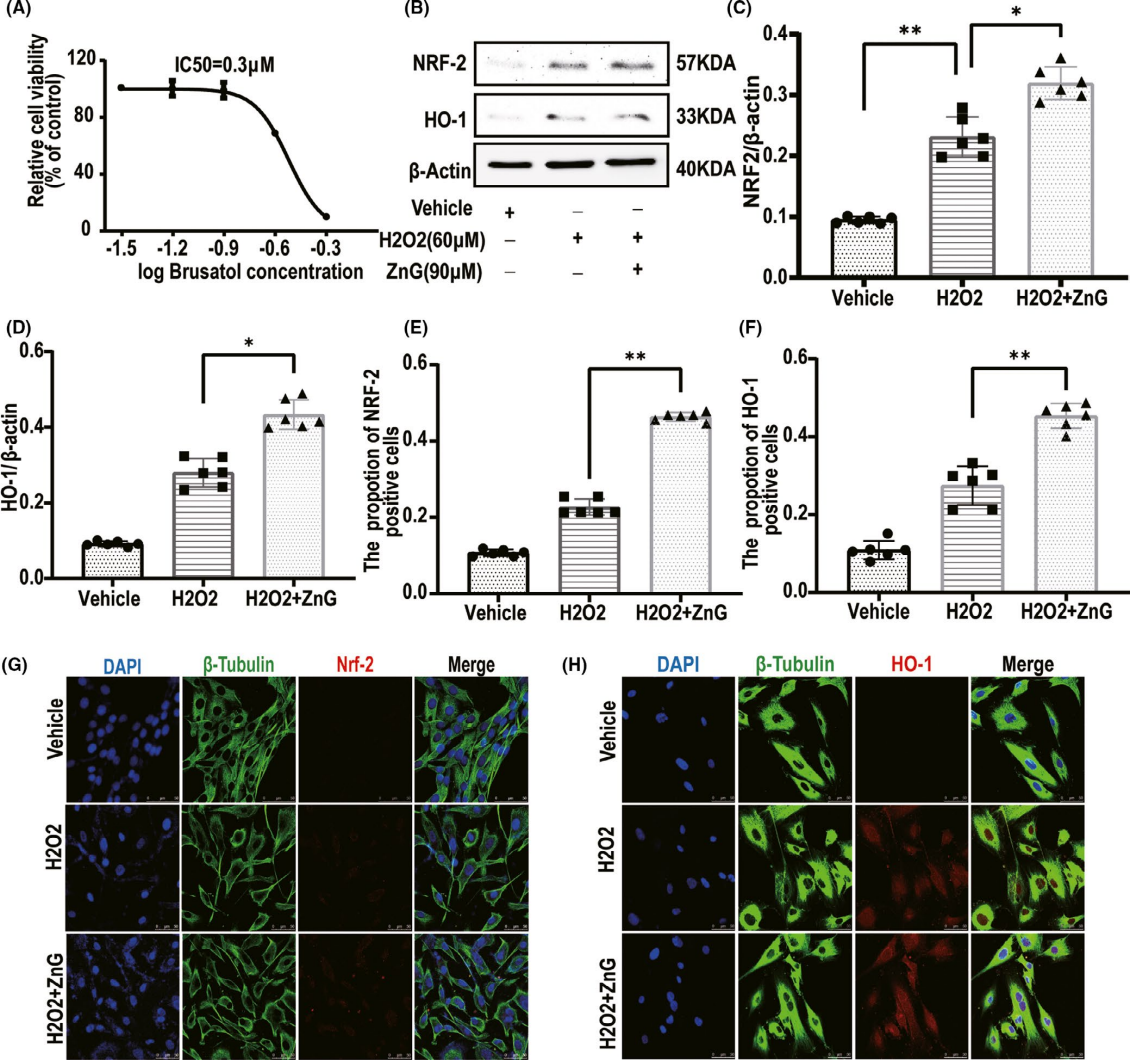
VSC4.1 can also increase the expression of NEF2/HO‐1 after zinc treatment to prevent H202. (A) The effects of different concentrations of Brusatol on the activity of VSC4.1 were determined by MTT assay (*n* = 6). (B) The expressions of NRF2 and HO‐1 were evaluated by Western blotting in VSC4.1 in each group (*n* = 6). (C,D) Quantification of NRF2 and HO‐1 expressions (*n* = 6, all the data are expressed as means ± SD, two‐way ANOVA followed by Tukey's post hoc test was applied). (E, F) Statistical analysis of immunofluorescence staining for positive expression of NRF2/HO‐1 in VSC4.1 from each group (*n* = 6, all the data are expressed as means ± SD, two‐way ANOVA followed by Tukey's post hoc test was applied) (G, H) Immunofluorescence staining was used to detect the level of NRF2/HO‐1 from each group (*n* = 6, scale bar =50 µm). * means *p* < 0.05, ** means *p* < 0.01, and *** means *p* < 0.001

The results of the MTT analysis showed that Brusatol has an IC50 of 0.3 μM (Figure 3A). The results of MTT H2O2 toxicity tests suggested that an H202 concentration was greater than 60 μmol, the survival rate of VSC4.1 was significantly inhibited, and cell viability was reduced to 50% (Figure S1A). MTT analysis was used to confirm the protective effect of zinc on VSC4.1 (Figure S1B). As mentioned above, treatment with excessive zinc can cause certain cytotoxicity. When the zinc concentration was greater than 100 μmol, the survival rate of VSC4.1 was significantly inhibited by (Figure S1B). Using Western blotting, we discovered the role of ZnG in VSC4.1 (Figure 3B–D). The expression of NRF2 in the ZnG + H2O2 group was significantly higher than that in the vehicle group and the H2O2 group. Therefore, we also found that ZnG markedly increased the expression of HO‐1 by promoting the expression of NRF2. We then used immunofluorescence to explore the expression levels of NRF2 and HO‐1 (Figure 3E–H). Using a confocal laser scanning microscope, we found that VSC4.1 treated with ZnG expressed NRF2 and HO‐1; the number of cells positively staining for these proteins was greater in the ZnG group than in the H2O2 group.

### 3.4 Zinc can save damage to mitochondria by improving the accumulation of oxidation products

**FIGURE 4:**
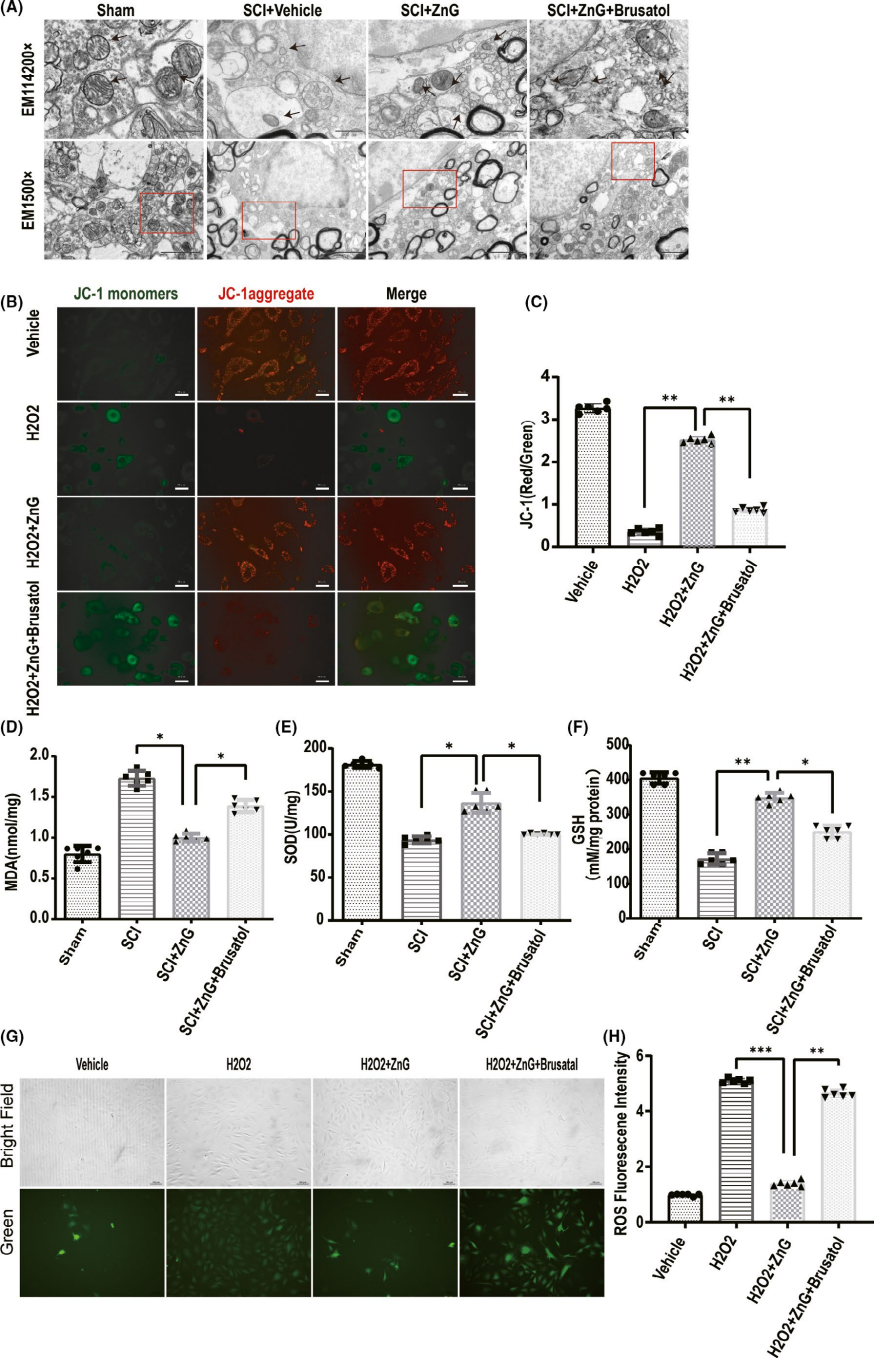
Zinc can save damage to mitochondria by improving the accumulation of oxidation products. (A) In the sham group, the mitochondria (arrow) were normal, and the ridges were clear; in the SCI group, the mitochondria were lost, and the mitochondria were atrophy and morphologically disordered (arrows). In the zinc treatment group, zinc reversed neuronal iron death and restored the subcellular structure to near‐normal levels. However, SCI + ZnG + Brusatol reversed the effect of zinc (*n* = 6 per group, scale bar =2 µm and scale bar = 500 nm); (B, C) VSC4.1’s mitochondrial membrane potential, analyzed by JC‐1 staining and compared with the H202 group and Brusatol group (*n* = 6, scale bar = 50 µm, all the data are expressed as means ± SD one‐way ANOVA followed by Bonferroni post hoc test was used). (D, E, F) The concentration of SOD, MDA, and GSH in each group's spinal cord tissue (*n* = 6 per group). Comparison between H2O2 + vehicle group and H2O2 + ZnG group, and H2O2 + ZnG group + Brusatol group. (G,H) Four different treatments of VSC4.1, using DCFH‐DA to detect intracellular ROS production through immunofluorescence images, immunofluorescence images, the first row is bright field images, microscope magnification: 200×. The fluorescence intensity of ROS expression in VSC4.1 of each group was counted by immunofluorescence staining (*n* = 6, scale bar = 150 µm, all the data are expressed as means ± SD, two‐way ANOVA followed by Tukey's post hoc test was applied). * means *p* < 0.05; ** means *p* < 0.01; and *** means *p* < 0.001.

The damaged tissue was observed by scanning electron microscopy. After zinc treatment, mitochondria had regular morphology and less vacuolation, while the mitochondria of the SCI group and the inhibitor group had irregular morphology, more vacuolation, and mitochondrial membrane collapse (Figure 4A). Next, we used the JC‐1 probe to assess the mitochondrial membrane potential, and the analysis indicated that the mitochondrial membrane potential of the ZnG group was higher than that of the SCI group and the Brusatol group (Figure 4B, C). We tested the MDA and SOD contents of the injured mouse spinal cord. The SOD content in the ZnG group was higher than that in the SCI + vehicle group and the SCI + ZnG + Brusatol group after injury. The content of MDA showed the opposite trend: The ZnG group had significantly lower MDA content than the SCI + vehicle group and the SCI + ZnG + Brusatol group (Figure 4D, E). The glutathione analysis results showed that the glutathione content of the ZnG group was significantly higher than that of the SCI group and the Brusatol group (Figure 4F). These changes are due to ZnG enhancing the ability to resist oxidative stress after spinal cord injury. We observed the oxidative stress products, and the analysis showed that the fluorescence intensity of ROS in the ZnG group was much weaker than that in the SCI group and the Brusatol group (Figure 4G, H).

### 3.5 Zinc inhibits ferroptosis after SCI by activating the GPX‐4 signaling pathway

**FIGURE 5:**
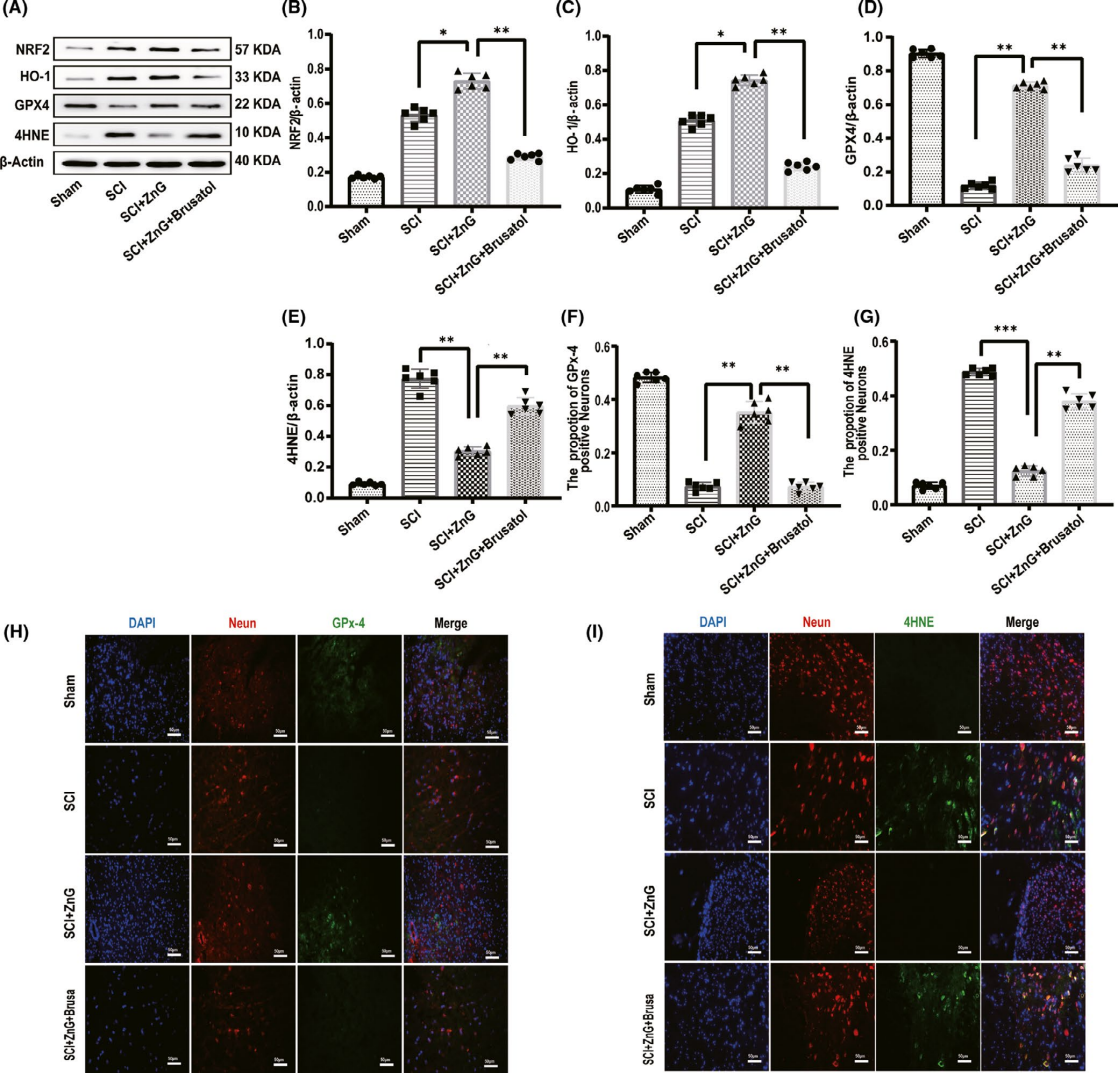
Zinc inhibits ferroptosis after SCI by activating the GPX‐4 signaling pathway. (A) The expressions of NRF2, HO‐1, GPX4, and 4HNE were evaluated at 3 d post‐SCI by Western blotting. (B–E) Quantification of NRF2, HO‐1, GPX4, and 4HNE expressions (*n* = 6, all the data are expressed as means ± SD, two‐way ANOVA followed by Tukey's post hoc test was applied). (F,G)Statistical analysis of immunofluorescence staining for positive expression of GPX4 and 4HNE in nerve cells from each group (*n* = 6, all the data are expressed as means ± SD, two‐way ANOVA followed by Tukey's post hoc test was applied). (H, I) Immunofluorescence staining was used to detect the level of GPX4/4HNE from each group (*n* = 6, scale bar = 50 µm). * means *p* < 0.05; ** means *p* < 0.01; and *** means *p* <0.001.

We injected mice with Brusatol for three days. The NRF2/HO‐1 ratio in the Brusatol group was lower than that in the ZnG group and the SCI group, and the specific inhibitor played a role (Figure 5A, B, C). Western blot detection of GPX4 and 4HNE found that the GPX4 content of the ZnG group was higher than that of the SCI group and the Brusatol group; the 4HNE content of the ZnG group was significantly lower than that of the SCI group and the Brusatol group (Figure 5A, D, E). Then, we used immunofluorescence staining. We found that the changes in GPX4 and 4HNE were in line with our hypothesis: The expression of GPX4 in the ZnG group was higher than that in the SCI group and the Brusatol group, while the expression of 4HNE was lower (Figure 5F–I). ZnG inhibited the ferroptosis pathway through upregulating the expression of NRF2, HO‐1, and GPX4 and inhibiting the lipid peroxidation (4HNE).

### 3.6 Zinc inhibits ferroptosis in VSC4.1 treated with H2O2, thereby reducing lipid peroxide levels

**FIGURE 6:**
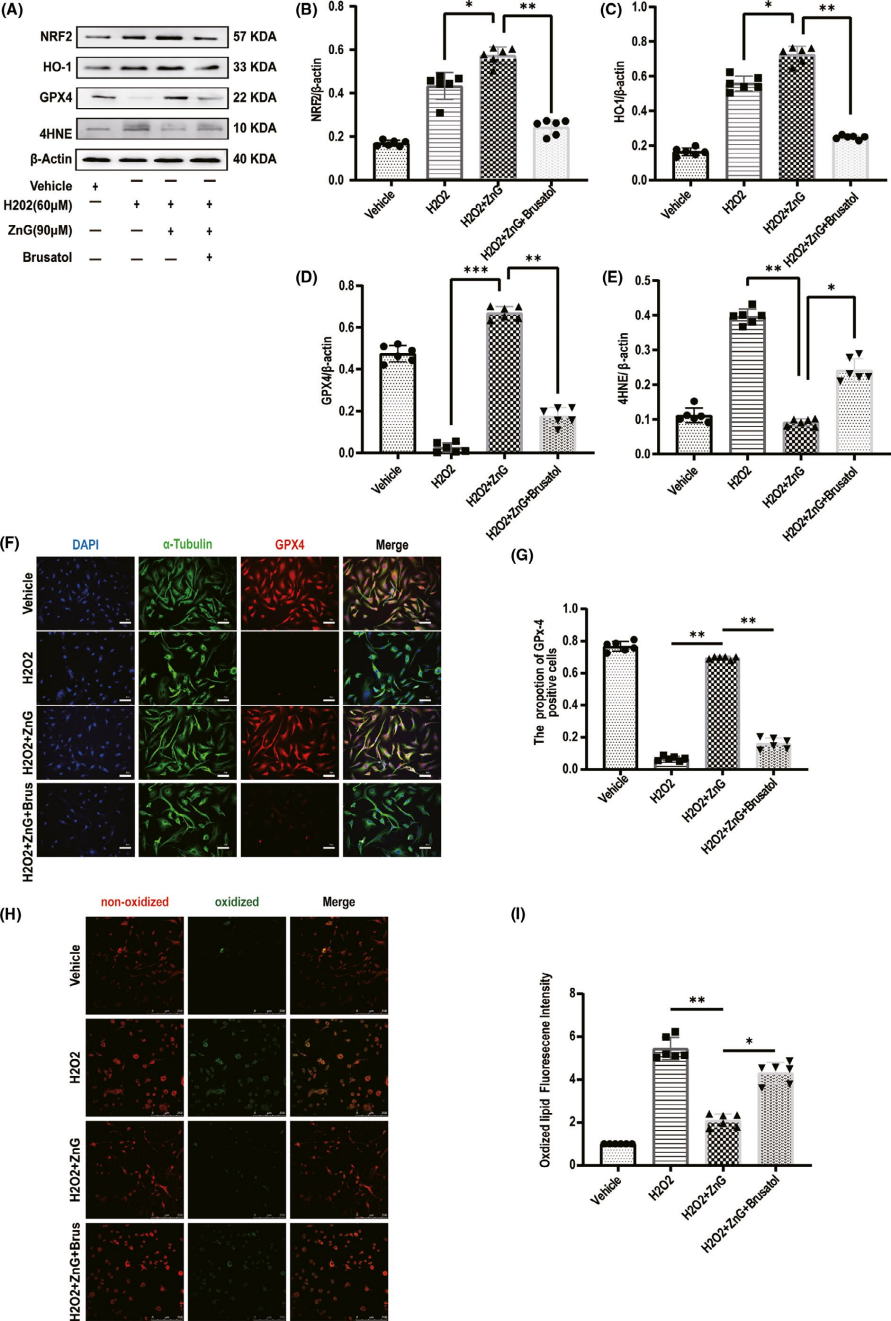
Zinc inhibits ferroptosis in VSC4.1 treated with H2O2, thereby reducing lipid peroxide levels. (A) The expressions of NRF2, HO‐1, GPX4, and 4HNE were evaluated by Western blotting in VSC4.1 in each group (*n* =6). (B‐E) Quantification of NRF2, HO‐1, GPX4, and 4HNE expressions (data shown as mean ± SEM, two‐way ANOVA with Tukey's post hoc test, *n* = 6). (F) Immunofluorescence staining was used to detect the level of GPX4 from each group (*n* = 6, scale bar = 50 µm). (G) Statistical analysis of immunofluorescence staining for positive expression of GPX4 in VSC4.1 from each group (*n* = 6, all the data are expressed as means ± SD, two‐way ANOVA followed by Tukey's post hoc test was applied). (H) Confocal microscopy showed the non‐oxidized lipid (red) and oxidized lipid (green) in VSC4.1 that were pretreated with ZnG (90 μmol/L) or not (*n* = 6, scale bar = 250 µm). (I) Statistical analysis of the fluorescence intensity of oxidized lipid expression in VSC4.1 from each group (*n* = 6, all the data are expressed as means ± SD, two‐way ANOVA followed by Tukey's post hoc test was applied). * means *p* < 0.05; ** means *p* < 0.01; and *** means *p* < 0.001

We simulated the oxidative stress damage environment in vitro. Western blot results showed that Brusatol had a more apparent inhibitory effect on NRF2 (Figure 6A–C). Western blot analysis found that the VSC4.1 in the ZnG group expressed more GPX4 than those in the H2O2 group and the Brusatol group (Figure 6A, D); immunofluorescence analysis showed that GPX4 expression was higher in the ZnG group than in the other two groups (Figure 6F, G). Western blot analysis also found that the 4HNE content of the ZnG group was significantly lower than that of the H2O2 group and the Brusatol group (Figure 6A, E). Moreover, we performed fluorescence detection of the lipid peroxide products of VSC4.1, and we observed that the content of lipid peroxide products after zinc ion treatment was lower in the ZnG group than in the H2O2 group and the Brusatol group (Figure 6H, I). In vitro, it was also found that ZnG treatment can up‐regulate the expression of NRF2, HO‐1, GPX4 and inhibit lipid peroxidation (4HNE), thereby inhibiting the occurrence of the ferroptosis.

### 3.7 Zinc can reduce the inflammation of damaged parts by inhibiting ferroptosis

**FIGURE 7:**
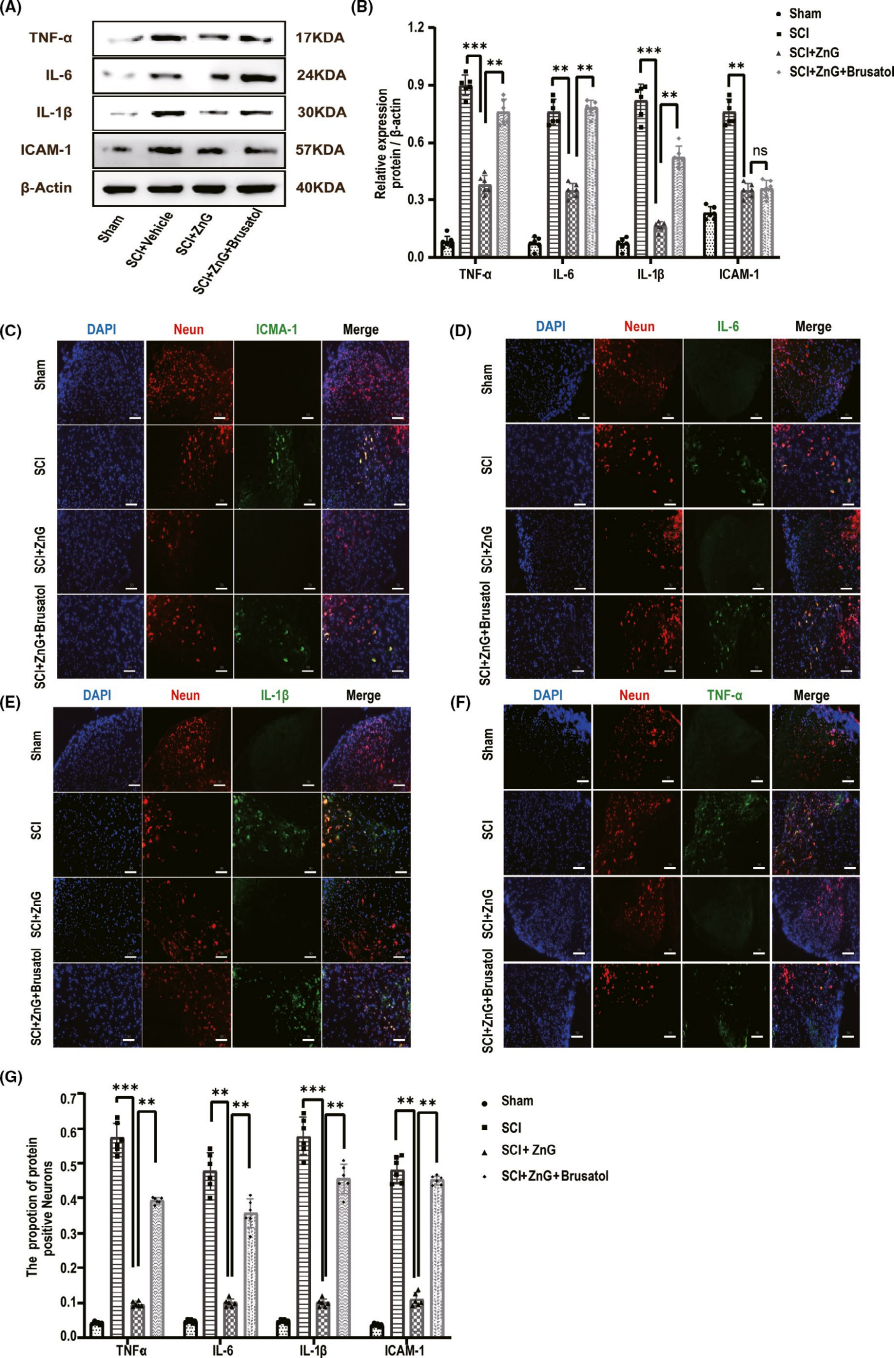
Zinc can reduce the inflammation of damaged parts by inhibiting ferroptosis. (A) The expressions of TNF‐α, IL‐6, IL‐1β, and ICAM‐1 were evaluated by Western blotting at 3 days post‐SCI in each group (*n* = 6). (B) Quantification of TNF‐α, IL‐6, IL‐1β, and ICAM‐1 expressions (*n* = 6, all the data are expressed as means ± SD, two‐way ANOVA followed by Tukey's post hoc test was applied). (C–F) Immunofluorescence staining was used to detect the level of TNF‐α, IL‐6, IL‐1β, and ICAM‐1 from each group (*n* = 6, scale bar = 50 µm). (G) Statistical analysis of immunofluorescence staining for positive expression of TNF‐α, IL‐6, IL‐1β, and ICAM‐1 in nerve cells from each group (*n* = 6, all the data are expressed as means ± SD, two‐way ANOVA followed by Tukey's post hoc test was applied). * means *p* < 0.05; **means *p* < 0.01; and *** means *p* < 0.001

**FIGURE 8:**
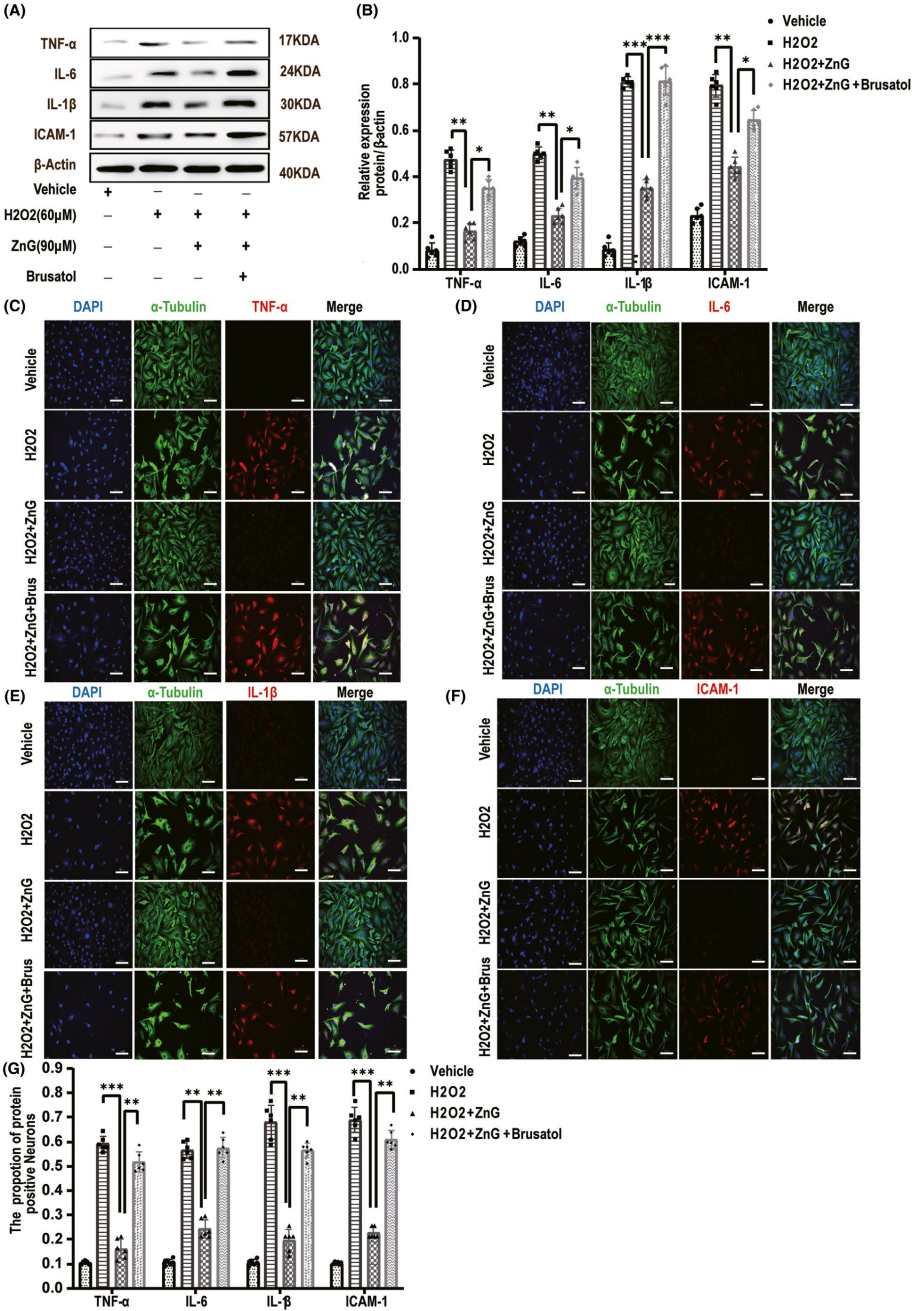
Zinc can reduce the inflammation of damaged parts by inhibiting ferroptosis. (A) The expressions of TNF‐α, IL‐6, IL‐1β, and ICAM‐1 were evaluated by Western blotting in VSC4.1 in each group (*n* = 6). (B) Quantification of TNF‐α, IL‐6, IL‐1β, and ICAM‐1 expressions (*n* = 6, all the data are expressed as means ± SD, two‐way ANOVA followed by Tukey's post hoc test was applied). (C–F) Immunofluorescence staining was used to detect the level of TNF‐α, IL‐6, IL‐1β, and ICAM‐1 from each group (*n* = 6, scale bar =50 µm). (G) Statistical analysis of immunofluorescence staining for positive expression of TNF‐α, IL‐6, IL‐1β, and ICAM‐1 in VSC4.1 from each group (*n* = 6, all the data are expressed as means ± SD, two‐way ANOVA followed by Tukey's post hoc test was applied). * means *p* < 0.05; **means *p* < 0.01; and *** means *p* < 0.001

Western blot analysis showed that the TNF‐α, IL‐6, IL‐1β, and ICAM‐1 levels were significantly increased at 3 days after spinal cord injury. Furthermore, after administration of the NRF2/HO‐1 pathway inhibitor Brusatol, the levels of these inflammatory factors significantly increased, while the inflammatory factors in the ZnG group were significantly lower than those in the SCI group and the Brusatol group (Figure 7A–B). We used immunofluorescence to identify the changes in these inflammatory factors and found that the expression of the inflammatory factors in the ZnG group was significantly lower than that in the SCI group and the Brusatol group (Figure 7C–G). In vitro Western blot experiments found that after ZnG treatment, these inflammatory factors had a low expression level, verifying our conclusions. After the inhibitor was added, the reduction of the inflammatory factors was inhibited; the same phenomenon was also observed with cellular immunofluorescence. Compared with those in the H2O2 group and the Brusatol group, the inflammatory factors in the ZnG group were significantly reduced (Figure 8A–G). ZnG inhibits programmed cell death and reduces the recruitment of inflammatory chemokines, thereby reducing the accumulation of inflammatory factors.

## 4 DISCUSSION

Ferroptosis is a recently discovered form of cell death.^6^ Injury sites produce a large number of oxidative stress products and inflammatory factors. These harmful products are suddenly produced in large quantities.^30^ If the body is unable to quickly clear or respond to these products, it will lead to the generation of more complicated derivative products that will cause further damage.

Zinc is an essential trace element in the human body that plays a crucial role in regulating the physiological and biochemical reactions.^31^ In previous studies, we found that zinc promotes the recovery of exercise behavior in mice with spinal cord injury.^16^, ^26^, ^28^, ^32^, ^33^ Recently, ferroptosis was discovered to be involved in spinal cord injury, many oxidation products, and hemoglobin fragmentation^30^; regrettably, the central nervous system is rich in unsaturated fatty acids, causing ferroptosis to play a pivotal role in the initial stage of injury.^34^, ^35^

Therefore, we should pay heed to the suppression of ferroptosis. Studies have shown that during spinal cord injury, the expression of GPX4 is damaged and the expression of 4NHE, a marker of lipid peroxides, is noticed to increase significantly, and death occurs in the acute phase of injury.^36^ In our experiments, after giving zinc gluconate, we found that the expression of NRF2/HO‐1 was considerably increased. Although their increase is a normal stress response of the body to injury, when we give zinc after ionization, Western blot and qRT‐PCR showed that the expression of NRF2/HO‐2 at the injured site was significantly higher than that of the control group. It was found that zinc has a strong regulatory effect on NRF2/HO‐1. We suppressed the occurrence of ferroptosis by controlling oxidative stress products. The most important morphological changes in ferroptosis of cells are changes in mitochondria: contraction of mitochondria, the collapse of mitochondrial cristae, changes in membrane potential, and rupture of the outer mitochondrial membrane.^37^, ^38^ After zinc treatment, the mitochondrial structure of damaged cells can be improved.

After zinc was administered, we detected ROS, SOD, MDA, and GSH in the spinal cord injury site and found that ROS were significantly reduced in zinc‐treated animals. Moreover, the SOD, MDA, and GSH contents of the SCI + vehicle group were significantly higher than those of the SCI + ZnG group. In a normal physiological state, these substances maintain a dynamic balance due to the removal of necrotic cells and metabolic waste. However, in an injured state, this balance is disrupted, and oxidative stress occurs.^39^ The products, namely necrotic cell debris, cannot be removed in time and damage other normal cells. Zinc accelerates ROS removal by promoting the synthesis of SOD and GSH to achieve a balanced state in the local microenvironment and prevent further cell damage.

In traumatic injuries in the central nervous system, the early inflammatory response greatly affects the prognosis of the injury.^40^

Cerebral ischemia‐reperfusion injury is closely linked to the inflammatory response, and it is also involved in spinal cord injury.^40^, ^41^, ^42^ Inflammation can be effectively controlled, and we believe the results will be surprising. Interestingly, it has been reported that GPX4 is involved in inflammation in a variety of traumatic conditions. As the active center of GPX4, glial cells in the cerebral cortex of mutant mice are activated after cysticercosis infection, leading to a proinflammatory phenotype change.

There are also reports claiming that when the GPX4 gene is knocked out, mouse spinal cord motor neurons disappear, keratinocytes are activated, and the mice show a paralyzed phenotype.^43^, ^44^ Programmed necrosis, especially of injured neuron cells, causes the release of a large amount of proinflammatory damage‐related molecules (damage‐associated molecular patterns, DAMPs) and inflammatory factors. These inflammatory factors attract and activate inflammatory cells, forming a cascade of inflammation.^45^

We were surprised to observe that after zinc was given to mice, inflammatory factors and intercellular adhesion factors showed a certain degree of downregulation. We established a model of oxidative stress‐related damage in vivo and observed downregulation of inflammatory factors; thus, we infer that the activation of GPX4 by zinc will inhibit the inflammatory response, thereby protecting surrounding neurons that are damaged.

Many points are still unclear. For example, zinc is an essential trace element, and we do not know whether it will affect other signaling pathways, such as the recently discovered critical FSP1‐CoQ‐dependent ferroptosis pathway (ROS‐Dependent Lipid Peroxidation and Reliant Antioxidant Ferroptosis‐Suppressor‐Protein 1 in Rheumatoid Arthritis).^46^ It is necessary to determine whether zinc plays a role in the regulation of this pathway. Further quantification of the effect at the injury site is also needed. Recent studies have discovered the protective mechanism of anti‐inflammatory after spinal cord injury. We speculate that zinc may also play roles on other cells in the central nervous system. For example, zinc can change the polarization of microglia and play an anti‐inflammatory effect. The protective effect of zinc on astrocytes inhibits demyelination damage.^47^ Zinc directly or indirectly affects the expression of related proteins, such as the regulation of the tumor necrosis factor receptor 2.^48^ The changes in some central nervous system cells and related protein factors in the acute phase of spinal cord injury can effectively affect the pathological changes of spinal cord injury and promote wound healing. Anti‐inflammatory environment, the more anti‐inflammatory and antioxidant effects of zinc require further experiments to verify these conjectures and perfect the mechanism of action of zinc.^26^ In general, it is clear that zinc is used as conventional antioxidants, and the clinical application of immune‐regulating trace elements provides a reliable theoretical basis and medication recommendations for protection against nerve injury.

## 5 CONCLUSION

Zinc promotes the degradation of oxidative stress products and lipid peroxides through the NRF2/HO‐1 and GPX4 signaling pathways to inhibit ferroptosis of neurons.

## CONFLICT OF INTEREST

The authors declare that there is no conflict of interest.

## AUTHOR CONTRIBUTIONS

M.G. completed the conception and design of the whole experiment. M.G, L.M, H.T, D.L, and J.L were involved in behavioral scoring and sample preparation. M.G, H.H, S.H, and C.Z. were involved in cell cultures. M.G. finished statistical analysis and manuscript preparation. Prof X.M. finished the final review and submitted the manuscript. All authors provided important intellectual content to the manuscript and approved to its publication.

## Supporting information

Fig S1

Supplementary Material

## Data Availability

The data that support the findings of this study are available from the corresponding author upon reasonable request. The data that support the findings of this study are available in the supplementary material of this article.
