# Supplementary figures and images for: Zinc attenuates ferroptosis and promotes functional recovery in contusion spinal cord injury by activating Nrf2/GPX4 defense pathway
Source: CNS Neurosci Ther. 2021 May 5;27(9):1023–40. doi: 10.1111/cns.13657 (PMC8339532; doi:10.1111/cns.13657)

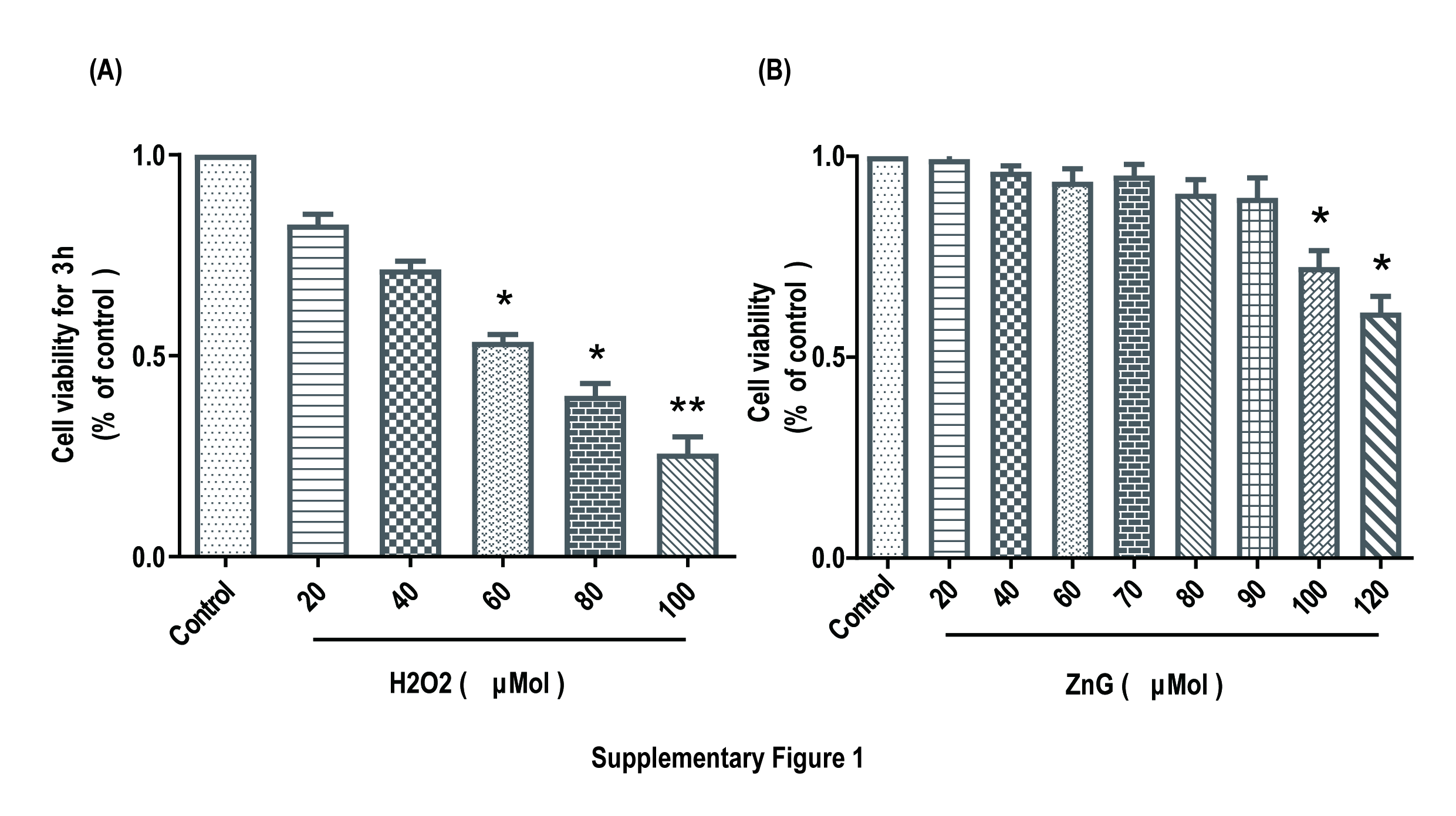

Supplement: Supplementary file 1 — Fig S1 [file CNS-27-1023-s001.tif]
