# Supplementary material for: Zinc attenuates ferroptosis and promotes functional recovery in contusion spinal cord injury by activating Nrf2/GPX4 defense pathway
Source: CNS Neurosci Ther. 2021 May 5;27(9):1023–40. doi: 10.1111/cns.13657 (PMC8339532; doi:10.1111/cns.13657)

Full unedited blot for Figure 1A HO-1

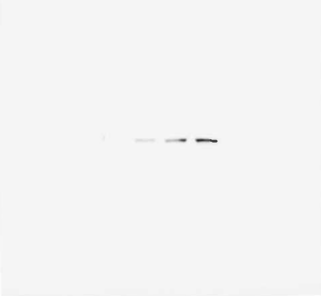

Full unedited blot for Figure 1A NRF-2

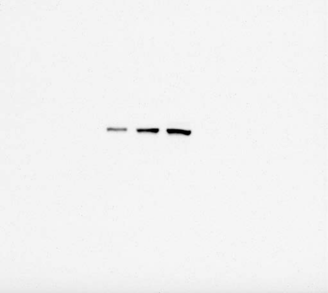

Full unedited blot for Figure 1A β-Actin

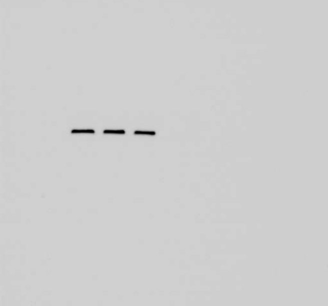

Full unedited blot for Figure 2B HO-1

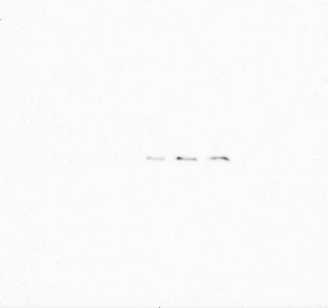

Full unedited blot for Figure 2B NRF-2

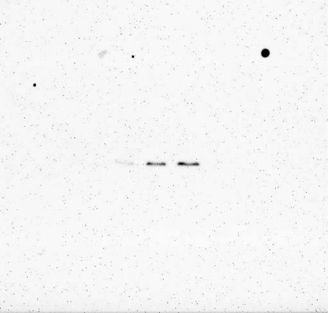

Full unedited blot for Figure 2B β-Actin

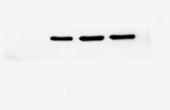

Full unedited blot for Figure 5A 4HNE

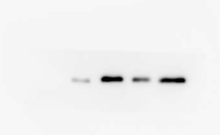

Full unedited blot for Figure 5A GPX4

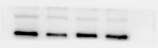

Full unedited blot for Figure 5A HO-1

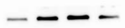

Full unedited blot for Figure 5A NRF-2

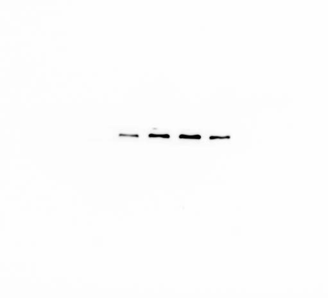

Full unedited blot for Figure 5A β-Actin

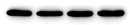

Full unedited blot for Figure 6A 4HNE

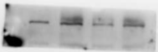

Full unedited blot for Figure 6A GPX4

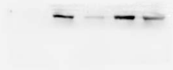

Full unedited blot for Figure 6A HO-1

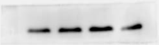

Full unedited blot for Figure 6A NRF2

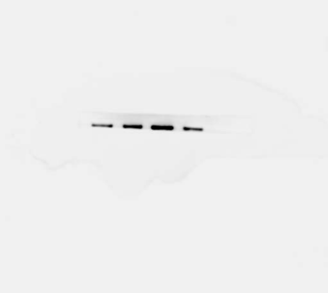

Full unedited blot for Figure 6A β-Actin

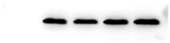

Full unedited blot for Figure 7A ICAM-1

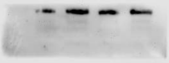

Full unedited blot for Figure 7A IL-1β

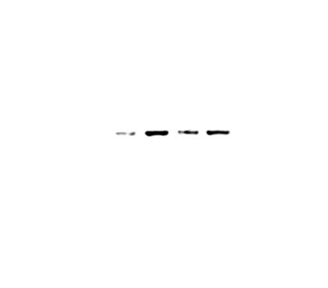

Full unedited blot for Figure 7A IL-6

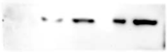

Full unedited blot for Figure 7A TNF-α

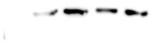

Full unedited blot for Figure 7A β-Actin

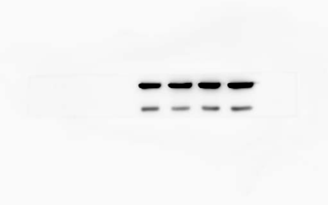

Full unedited blot for Figure 8A ICAM-1

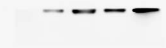

Full unedited blot for Figure 8A IL-1β

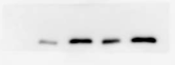

Full unedited blot for Figure 8A IL-6

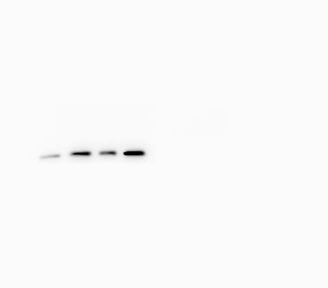

Full unedited blot for Figure 8A TNF-α

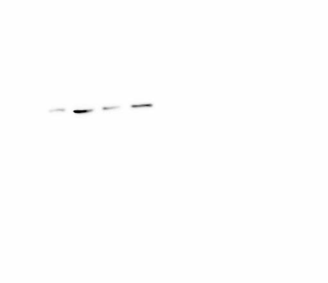

Full unedited blot for Figure 8A β-Actin

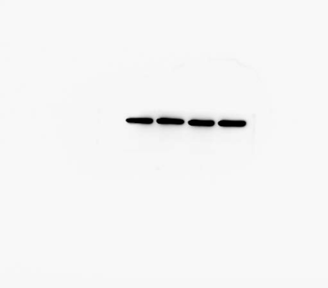

Supplement: Supplementary file 2 — Supplementary Material [file CNS-27-1023-s002.pdf]
